# The Management of Peripheral Ossifying Fibroma: A Case Presentation

**DOI:** 10.7759/cureus.70818

**Published:** 2024-10-04

**Authors:** Sneha Dare, Pavan Bajaj, Unnati Shirbhate, Shivani Thakre, Husna Tehzeeb

**Affiliations:** 1 Department of Periodontics, Sharad Pawar Dental College and Hospital, Datta Meghe Institute of Higher Education and Research, Wardha, IND; 2 Department of Oral Pathology and Microbiology, Sharad Pawar Dental College and Hospital, Datta Meghe Institute of Higher Education and Research, Wardha, IND

**Keywords:** histopathological evaluation, peripheral ossifying fibroma, reactive lesions, recurrence, surgical excision

## Abstract

One of the most frequent conditions found in the oral cavity includes reactive lesions. Peripheral ossifying fibroma (POF) is a prevalent condition characterized by an overgrowth, originating from the gingival tissue, periodontal ligament, and periosteum. The condition manifests as a small mucosal nodule and can be difficult to accurately identify since it resembles other lesions. Chronic irritation and trauma are considered to be etiological agents. It is more prevalent in females and typically impacts the anterior region of the maxilla and mandible. The recommended therapy for POF involves surgical excision and eliminating any factors that may be causing irritation in the affected area. It can grow significantly if not surgically removed early. Treatment is necessary to prevent recurrence. It can be misdiagnosed due to overlapping features with other lesions. Correct biopsy technique and histopathological evaluation are crucial for accurate diagnosis. This case report illustrates the management of gingival overgrowth in an 18-year-old female patient after comprehensive clinical evaluation by using conventional surgical excision with the help of a scalpel. The final diagnosis of POF was confirmed using histopathologic evaluation. The treatment led to favorable outcomes in the patient regarding mastication, speech, and esthetics.

## Introduction

Gingiva often develops localized overgrowths, which are typically described as reactive lesions. Several of these lesions can be recognized as distinct entities based on their characteristics and consistent histomorphology [1]. Since the late 1940s, the literature has documented the presence of intraoral ossifying fibromas. Peripheral ossifying fibroma (POF) is one of the most prevalent forms of fibro-osseous lesions [2]. It is a type of gingival overgrowth that is made up of a stroma of cellular fibroblastic connective tissue and is linked to the production of randomly distributed foci of mineralized product, which can be dystrophic calcification, cementum-like tissue, or bone [3]. It primarily affects young individuals and appears to have its origin in periodontal ligament cells [4]. POF typically presents as a small, distinct, localized mass with a base that is either pedunculated or sessile on the gingival margin. The dimensions of the lesion typically range from less than 1.5 cm in diameter, while larger ones may occasionally be observed [5]. The color can range from a light rosy shade to a deep cherry red. Moreover, the lesions tend to occur more frequently in females and have a high likelihood of recurring despite being a benign reactive growth [6].

POF lesion typically arises as a result of trauma or local irritants due to the presence of calculus, plaque, microorganisms, and ill-fitting prosthetic or orthodontic appliances. The radiological characteristics of the POF appear to differ amongst instances. Typically, radiographs do not reveal any visible signs of underlying bone involvement. Occasionally, there have been observations of minor bone resorption [7]. Due to the similarities in clinical presentation among pyogenic granuloma, peripheral giant cell granuloma, and oral irritation fibroma, a biopsy is often necessary for accurate diagnosis. POF management involves removing local irritants through rigorous plaque control and surgical removal of excessive tissue growth. The conventional treatment strategy includes surgical excision followed by a biopsy of the lesion [8]. The mineralized components in POF exhibit a wide range of 23% to 75%. Buchner and Hansen identified three different components in POF: bone (woven/lamellar), dystrophic calcifications, and cementum [1]. Under microscopic examination, these proliferations consist of poorly circumscribed fibrous tissue composed of spindle-shaped cells. These cells do not exhibit any abnormal characteristics, and their distinguishing feature is the synthesis of bone (either mature or immature), cementum, or calcifications in varying proportions [9]. This case report represents the management of POF using scalpel surgical excision and its histopathologic presentation.

## Case presentation

**Figure 1:**
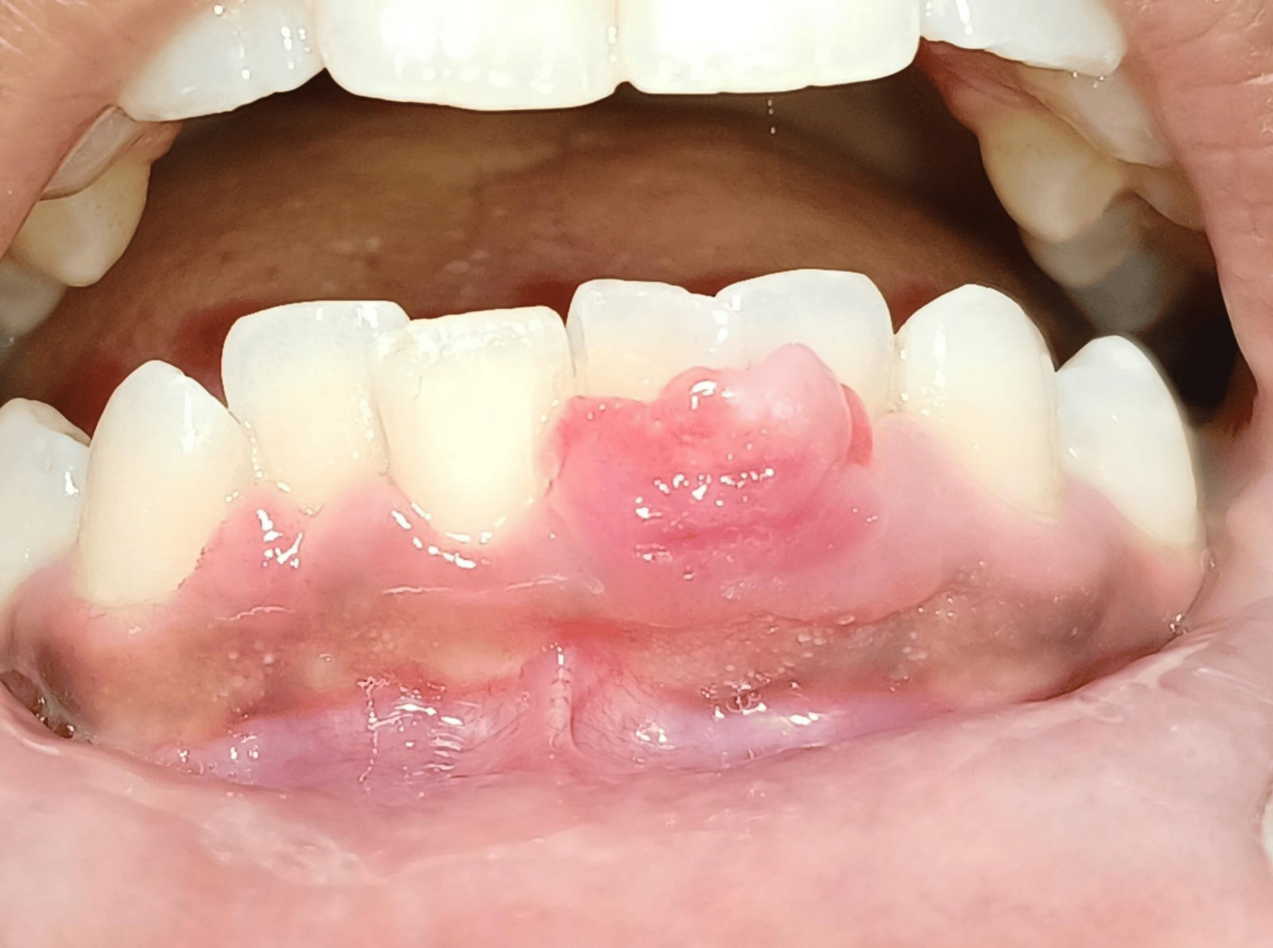
Preoperative view POF lesion extending mesial aspect of 41 to the mid-buccal region of 32 POF - peripheral ossifying fibroma

An 18-year-old female reported to the Department of Periodontics with a complaint of a progressively enlarging overgrowth in the lower anterior region of the jaw. She mentioned that the growth initially appeared as a little nodule and kept on increasing in size over the past month. The lesion was asymptomatic and occasionally bled when subjected to trauma from a toothbrush, and it was causing interference with occlusion. It was present in the buccal aspect of the extending from the mesial aspect of 41 to the mid-buccal region of 32 with no involvement in the lingual aspect. It was irregular in shape, pale pink in color, non-tender, fibrous, and firm in consistency, and had a sessile base, which can be appreciated in Figure 1. 

**Figure 2:**
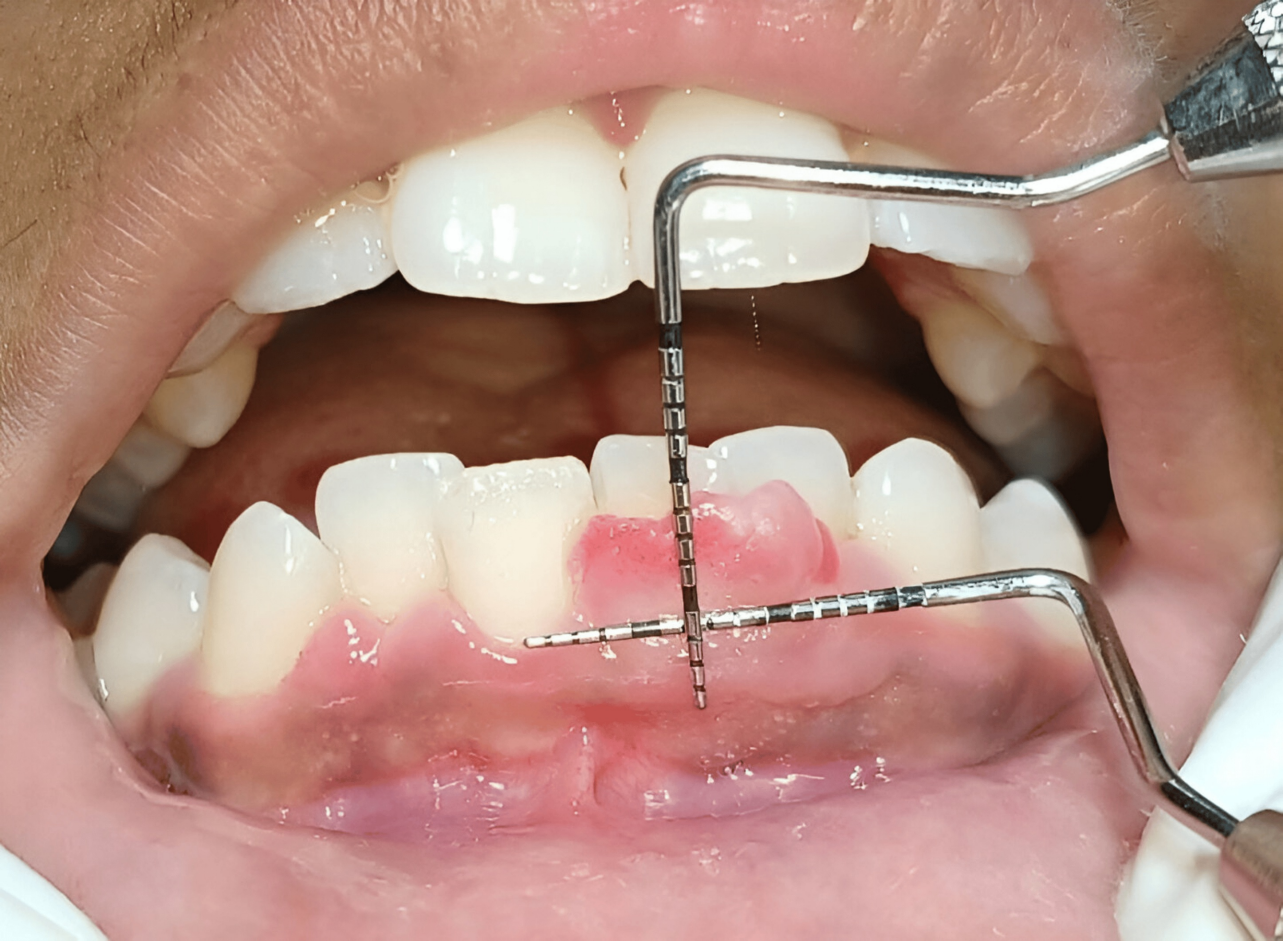
Measurements of POF lesion using University of North Carolina (UNC)-15 probe depicting the size to be 10x8 mm POF - peripheral ossifying fibroma

The dimensions of the lesion were 10x8 mm, which were measured using a University of North Carolina (UNC)-15 probe, which can be seen in Figure 2. No radiological signs of involvement of the alveolar ridge were observed.

**Figure 3:**
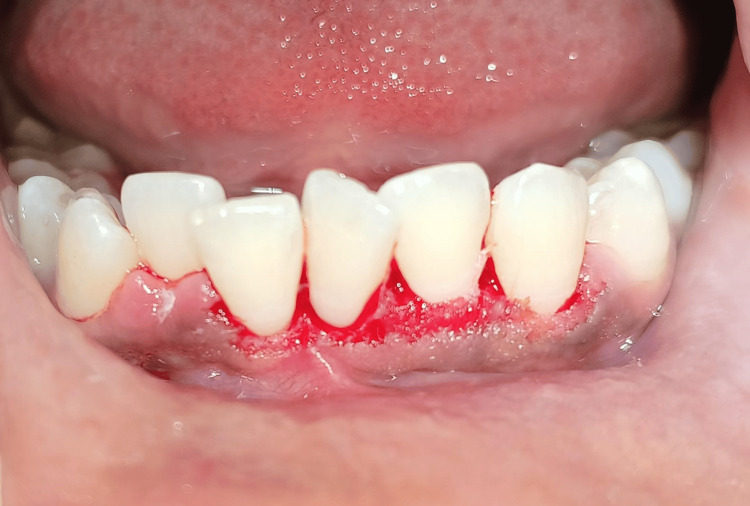
Surgical excision was done along with thorough curettage of the adjacent periodontal tissue

There was no significant medical history or familial history. Following a routine hematological examination, the findings were within an accepted range. Based on clinical presentation, the provisional diagnosis of peripheral giant-cell granuloma was made with a differential diagnosis of traumatic fibroma and POF. After proper counseling, the patient provided written informed consent for the surgical treatment. As seen in Figure 3, under local anesthesia, the overgrowth was surgically removed using a No. 15 scalpel blade, and the surrounding periodontium was carefully curettaged to prevent recurrence.

**Figure 4:**
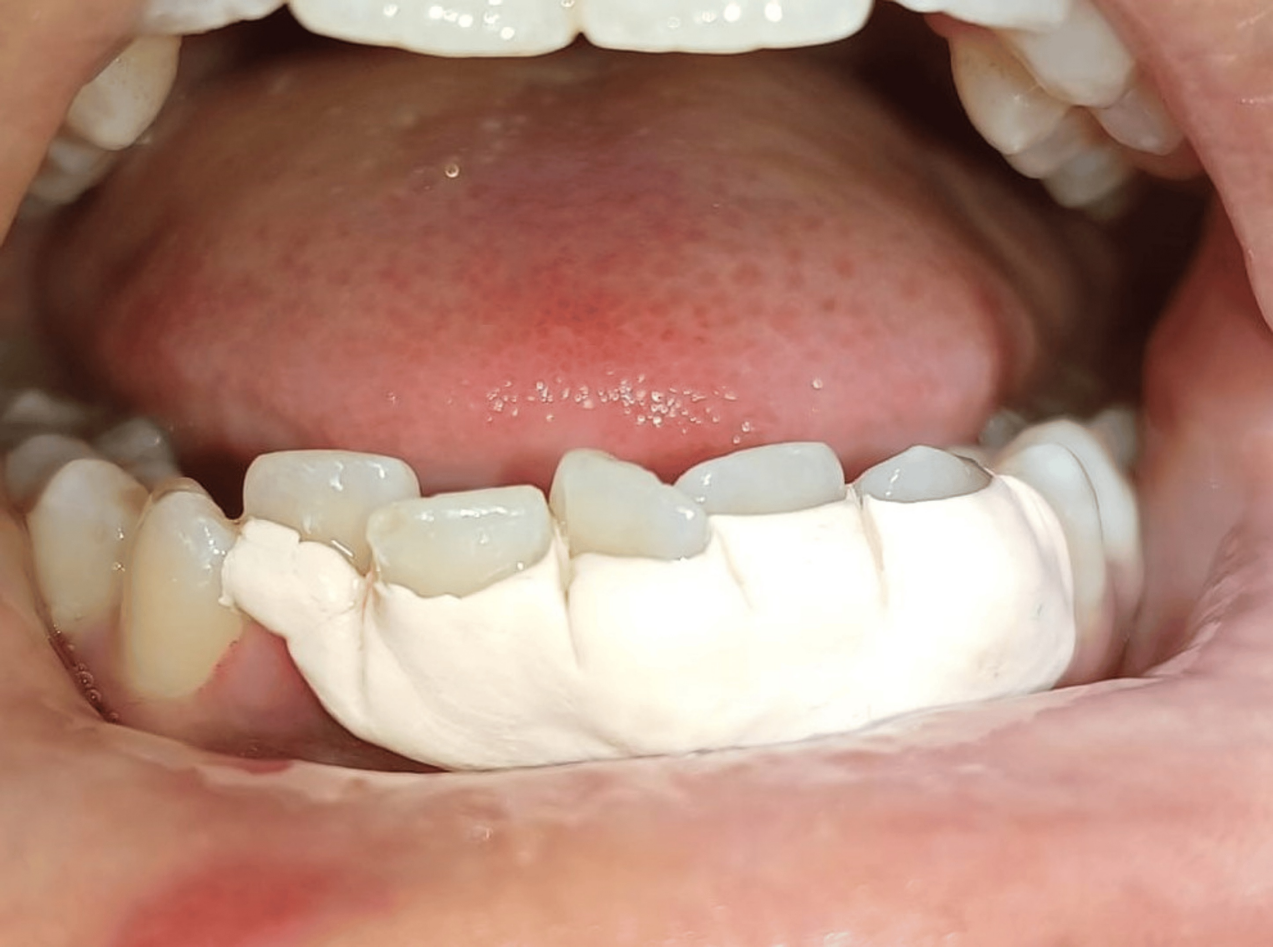
Application of periodontal dressing after procedure to protect the surgical site and improve healing

Figure 4 shows an application of periodontal dressing (COE-PAK; GC International AG, Luzern, Switzerland) on an operated area to protect the surgical site and facilitate healing by helping maintain proper oral hygiene. No antibiotics were prescribed to the patient preoperatively/postoperatively. 

**Figure 5:**
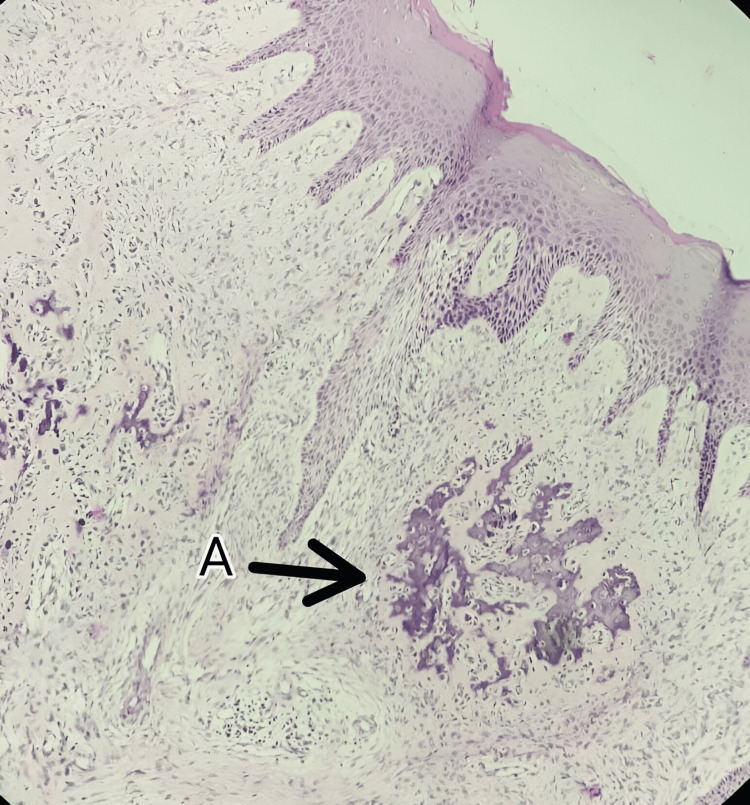
Histopathological section of excised tissue under low magnification revealed features of POF A) foci of calcification POF - peripheral ossifying fibroma

The excised tissue was sent for histopathological examination. Figure 5 is the histopathologic slide where the Hematoxylin and Eosin (H&E) stained section shows stratified squamous epithelium and fibrocellular connective tissue stroma. Areas of dystrophic calcification are appreciated in the connective tissue adjacent to the epithelium, where histopathological features suggest POF. 

**Figure 6:**
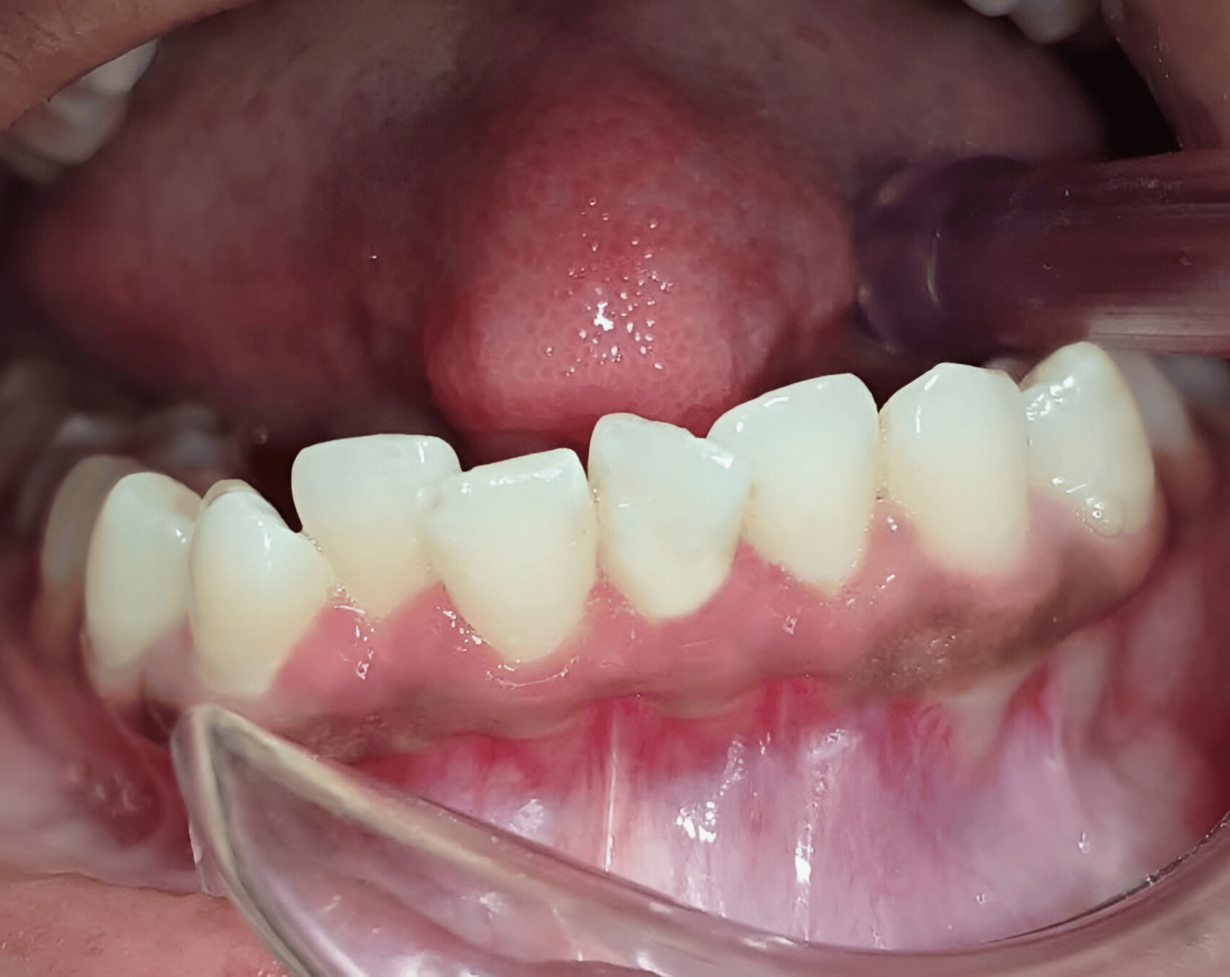
Postoperative site at one month follow-up visit demonstrating uneventful healing

The patient was reviewed after one week, followed by one month after the surgical procedure. Figure 6 shows the postoperative view at one-month follow-up. The healing was uneventful, and the improvement of aesthetics can be appreciated. No instances of recurrence were observed up to the recent follow-up of six months postoperatively.

## Discussion

Individuals with POF may encounter various challenges, such as esthetic concerns and occlusal disturbances; in severe cases, there will be displacement of teeth, resulting in functional issues and difficulties in maintaining optimal oral hygiene. Although POFs are generally painless, a few patients might encounter minor bleeding during gentle probing and some discomfort caused by the size and location of the lesion [10]. They can enlarge and obstruct the normal process of chewing and ingesting if left untreated. Therefore, it is imperative to implement early diagnosis and prompt treatment [11]. The higher frequency of POF among women, the growing occurrence in the second decade, and the reduced incidence following the third decade point to hormonal effects may be involved [6]. Radiographically, in some cases, POF may not reveal any appreciable alterations. Still, depending on the degree of mineralization, certain patients display different radiodensity inside the lesion [9]. A thorough treatment approach is advisable for POFs due to the high recurrence rate of 8-20%. The approach should include complete removal of the lesion along with debridement of underlying bone and tooth. The likelihood of POF recurrence could be attributed to the remaining fragments of the lesion, the continued presence of local irritants, or repetitive damage [12]. 

There are various treatment modalities present for POF. Using the laterally displaced flap technique, Assimi et al. in their case report showed the successful surgical excision and periodontal management of POF. They underlined the need to quickly correct residual gingival defects to avoid aesthetic and functional discrepancies [8]. In their case report, Gulati et al. demonstrate the efficacy of a diode laser in effectively managing significant postoperative bleeding. The authors highlight the laser's ability to achieve hemostasis and prevent excessive bleeding after excision [13]. The POF in the case report by Nadimpalli et al. was surgically excised, resulting in the lesion's eradication and the prevention of its growth and possible harm to the surrounding tissues. After five months, there was no recurrence of POF, suggesting that surgical excision successfully stopped lesion regrowth [14]. Hence, local excision is the most commonly reported mode of treatment. To reduce the risk of recurrence, the affected periodontal ligament and periosteum must be completely excised. Due to the high recurrence rate of POF, close postoperative monitoring and follow-up are essential. Patients should be followed up annually to detect any recurrences early. 

To prevent the recurrence of POF, several key strategies should be implemented following surgical excision of the lesion. The most critical factor in preventing recurrence is the complete excision, which includes not only the visible lesion but also any surrounding tissue that may harbor remnants of the growth. Identifying and addressing any local irritants is essential. After treatment, patients should have regular follow-up appointments to monitor for any signs of recurrence. This allows for early detection and intervention if the fibroma begins to reappear. Patients should be informed about the nature of POF, its potential for recurrence, and the importance of maintaining good oral hygiene practices. This includes brushing, flossing, and possibly using antimicrobial mouth rinses to keep the area clean. Surgical excision remains the treatment of choice for POF, with careful attention to complete removal and management of local irritants to reduce recurrence rates. In this case, a follow-up period of six months demonstrated successful outcomes without any signs of relapse, underscoring the importance of thorough surgical technique and postoperative monitoring in managing POF effectively. Complete case evaluation is crucial before planning a treatment. This case lacks the ability to provide radiographic presentation and the follow-up time is also short. 

## Conclusions

This case report presented the surgical treatment of overgrowth in the mandibular anterior region that was histologically identified as POF, demonstrating improvement in both functional and aesthetic considerations. Early diagnosis and conservative care are critical in such lesions since they can become more destructive over time if not treated. The most accepted treatment procedure consists of surgical excision followed by histopathologic examination and follow-up. Due to its high recurrence rate, a regular follow-up is essential following excision.
